# Treatment of Detached Retina by Iodine Injections into the Bulb

**Published:** 1890-11-01

**Authors:** 


### TREATMENT OF DETACHED RETINA BY IODINE INJECTIONS INTO THE BULB.

At a recent meeting of the Berlin Medical Society, Dr. Schaeler read a report on twenty-six cases that he had treated by injections. In two he had used a solution of sodium salicylate (1 in 3), in two Lugol's iodine solution, and in the remaining twenty-two the tincture of iodine. In four a cure was hopeless, though partial adhesion and subsidence of the inflammation followed. Three cases failed through hæmorrhage, and one in consequence of an accident. In seven the attachment was only temporary, and in two others, in which the reactive inflammation was too slight to effect a lasting adhesion, the operation had been repeated with good promise of success; while another of the like character had been cured by the second operation. Two of the unsuccessful cases were treated with the salicylate, and one with Lugol's solution; but deducting these, as well as the four in which success was not to be expected, the result had been six cases of perfect reposition and two which give a prospect of cure, out of seventeen treated with tincture of iodine. Considering the grave character of the lesion, these results are very encouraging.

